# An In Vitro Approach for Investigating the Safety of Lipotransfer after Breast-Conserving Therapy

**DOI:** 10.3390/jpm12081284

**Published:** 2022-08-05

**Authors:** Theresa Promny, Chiara-Sophia Kutz, Tina Jost, Luitpold V. Distel, Sheetal Kadam, Rafael Schmid, Andreas Arkudas, Raymund E. Horch, Annika Kengelbach-Weigand

**Affiliations:** 1 Department of Plastic and Hand Surgery, University Hospital Erlangen, Friedrich-Alexander-Universität Erlangen-Nürnberg, 91054 Erlangen, Germany; 2 Department of Radiation Oncology, University Hospital Erlangen, Friedrich-Alexander-Universität Erlangen-Nürnberg, 91054 Erlangen, Germany

**Keywords:** adipose-derived stem cells, ADSC, MCF-10A, mammary epithelial cells, irradiation, fibroblasts, epithelial-mesenchymal transition, mesenchymal markers

## Abstract

The application of lipotransfer after breast-conserving therapy (BCT) and irradiation in breast cancer patients is an already widespread procedure for reconstructing volume deficits of the diseased breast. Nevertheless, the safety of lipotransfer has still not been clarified yet due to contradictory data. The goal of this in vitro study was to further elucidate the potential effects of lipotransfer on the irradiated remaining breast tissue. The mammary epithelial cell line MCF-10A was co-cultured with the fibroblast cell line MRC-5 and irradiated with 2 and 5 Gy. Afterwards, cells were treated with conditioned medium (CM) from adipose-derived stem cells (ADSC), and the effects on the cellular functions of MCF-10A cells and on gene expression at the mRNA level in MCF-10A and MRC-5 cells were analyzed. Treatment with ADSC CM stimulated transmigration and invasion and decreased the surviving fraction of MCF-10A cells. Further, the expression of cytokines, extracellular, and mesenchymal markers was enhanced in mammary epithelial cells. Only an effect of ADSC CM on irradiated fibroblasts could be observed. The present data suggest epithelial–mesenchymal transition-like changes in the epithelial mammary breast cell line. Thus, the benefits of lipotransfer after BCT should be critically weighed against its possible risks for the affected patients.

## 1. Introduction

Multimodal treatment of breast cancer consists of surgical treatment as well as systemic therapies and adjuvant radiation, depending on the underlying breast cancer subtype and breast cancer stage. Thereby, surgical therapy has become more conservative over the last decades and breast-conserving therapy (BCT), usually followed by radiation therapy, has evolved into a widespread alternative to mastectomy for patients with early breast cancer [1,2]. However, BCT might provide disfiguring results, so patients and surgeons aim to reconstruct the original anatomical contours of the breast [3,4,5]. This can be achieved by lipofilling or lipotransfer, a procedure in which fat is harvested via liposuction from typically used donor-sites (e.g., the abdomen, flank, thighs) and subsequently transplanted into the breast. Besides its many advantages including a natural appearance and texture, low donor-site morbidity, and easy availability, autologous fat transplants often show a low rate of graft survival [6]. The fat grafts include a minor fraction of adipose-derived stem cells (ADSC). To ameliorate graft viability, supplementation of the fat grafts with ADSC has been proposed [7,8]. ADSC have immunomodulatory and paracrine characteristics and secrete several cytokines, chemokines, and growth factors, e.g., vascular endothelial growth factor (VEGF), fibroblast growth factor 2 (FGF-2), and insulin-like growth factor 1 (IGF-1) [9,10,11]. These capacities are associated with regenerative effects and might contribute to higher fat engraftment, especially in irradiated tissues [12]. On the other hand, they might confer an oncological potential on the grafted fat. The issue of the oncological safety of lipotransfer, particularly to a site where malignancy was treated, has been discussed widely and controversially in the literature. Whereas predominantly clinical studies claim a safe use of lipotransfer [13,14,15,16,17,18], pre-clinical studies suggested an ADSC-induced promotion of breast cancer growth and tumor angiogenesis [9,19,20,21,22]. Among other factors, the role of vascularization in any recipient tissue for cell ingrowth or proliferation is certainly relevant [23,24,25]. Further, previous studies showed that ADSC can also influence healthy breast tissue and induce an epithelial-to-mesenchymal transition in mammary epithelial cells [26]. This observation should not be neglected, as many patients undergo lipotransfer after BCT with the remaining tissue consisting partially of mammary epithelial cells. In most of the cases, this tissue has also been irradiated. Radiotherapy has an outstanding role in the treatment of breast cancer to eliminate (residual) cancer cells. However, it has also an effect on the surrounding tissue. There is evidence that the microenvironment contributes to the initiation, proliferation, and metastasis of a tumor [27,28,29]. Thereby, fibroblasts, the predominant cellular component of the tumor microenvironment, play a decisive role. Previous investigations showed that stromal fibroblasts are involved in the control of the growth and morphogenesis of normal and tumorigenic breast epithelial cells [30,31]. Additionally, they can be activated by endogenous stimuli, such as cytokines and growth factors secreted by tumor cells, or by external stimuli, such as radiation therapy [32,33,34]. Those activated “cancer-associated fibroblasts” (CAF) are considered fundamental actors in tumor progression and were shown to promote tumor growth, invasion, and metastasis [35]. Further, they have been attributed a role in stroma-mediated radiotherapy resistance [36]. Thus, tumor development, growth, recurrence, and resistance to therapies is dependent on various factors. When investigating the oncological safety of lipotransfer after BCT, the interplay of different cell types and external stimuli should be considered.

The present experimental in vitro study aims to further elucidate the effect of lipotransfer on breast tissue. Therefore, we investigated the influence of ADSC on mammary epithelial cells after exposure to irradiation while taking into account an important element of the microenvironment, the fibroblasts, by using a co-cultivation model. Thereby, functional analysis was performed, and alterations in gene expression profiles were investigated.

## 2. Materials and Methods

### 2.1. Cell Lines and Cell Culture

MCF-10A (ATCC^®^ CRL-10317™), a human mammary epithelial cell line, was a kind gift from Matthias Rübner, Department of Obstetrics and Gynaecology, University Hospital Erlangen. MCF-10A cells were cultivated in Mammary Epithelial Cell Growth Medium (MECGM; PromoCell GmbH, Heidelberg, Germany) supplemented with 5 µg/mL insulin, 0.5 µg/mL hydrocortisone, 10 ng/mL epidermal growth factor (EGF), 0.004 mL/mL bovine pituitary extract (BPE; PromoCell), 100 ng/mL cholera toxin (Sigma Aldrich, St. Louis, MO, USA), and 1% penicillin/streptomycin (Sigma Aldrich). 

The human fibroblast cell line MRC-5 was purchased from ATCC (ATCC^®^ MRC-5 CCL-171™). It was cultivated in Eagle’s Minimum Essential Medium (EMEM; ATCC, Manassas, VA, USA) supplemented with 10% fetal calf serum superior (FCS superior; Sigma Aldrich). ASC/TERT1, a human-adipose-tissue-derived telomerase-immortalized mesenchymal stem cell line, was purchased from Evercyte (Evercyte GmbH, Vienna, Austria). For their cultivation we used the Endothelial Cell Growth Medium (EGM)-2 BulletKit™ (Lonza Group AG, Basel, Switzerland), a culture system containing Endothelial Cell Basal Medium-2 (EBMTM-2) and EGMTM-2 SingleQuotsTM Supplements, enriched with 200 µg/mL Geneticin™ (Gibco^®^ Life Technologies, Carlsbad, CA, USA) and 2% FCS superior (Sigma Aldrich). All cells were cultivated at 37 °C and 5% CO_2_. The medium was changed every 2–3 days.

### 2.2. Experimental Setup

A total of 2 × 10^4^ MCF-10A cells were seeded in their standard medium into the lower compartment of a 6-well transwell system (pore size 0.4 µm) and cultivated until 50–60% confluency. Subsequently, the medium was replaced by a mixed co-culture medium consisting of 2/3 MEGM and 1/3 EMEM, including their respective supplements, and 6 × 10^5^ MRC-5 cells were placed into the transwells (6-Well ThinCert™ Cell Culture Inserts, Greiner Bio-One, Frickenhausen, Germany). For the control group, MCF-10A cells were seeded into the transwells. Irradiation with 0 (control), 2, and 5 Gy was applied after 24 h of co-cultivation. After another 24 h, co-cultures were either treated with ADSC conditioned medium (CM) for 48 h for later RNA isolation and flow cytometry or irradiated co-cultures were cultivated for a further 48 h with a mixed co-culture medium. Thereafter, MCF-10A cells were collected for later transmigration and invasion analysis with ADSC CM.

For an analysis of gene expression of MRC-5 cells, MRC-5 cells in the transwells were harvested for RNA isolation. For monoculture control, 4 × 10^3^ MRC-5 cells were seeded in the lower compartment and cultivated until 50–60% confluency. The further procedure was analogous to the setup described above.

### 2.3. Preparation of ADSC Conditioned Medium (CM)

ASC/TERT1 were seeded in 75 cm² cell culture flasks with EBM-2 medium containing standard supplements. They were cultivated until they reached 90% confluency. After washing the cells with phosphate-buffered saline (PBS; Sigma Aldrich), ADSC were incubated in EBMTM-2 without supplements for 24 h. Filter tubes (Amicon^®^ Ultra-15 Centrifugal Filter Devices, 3 K; Merck KGaA, Darmstadt, Germany) were used for concentrating CM. CM was centrifuged at 4000× *g* for 30 min, and the ultrafiltrate of 15 mL CM was diluted with 2/3 MECGM containing 5% supplement mix and 1/3 EMEM supplemented with 0.5% FCS superior (in the following described as “reduced mixed co-culture medium”) up to a total volume of 5 mL to obtain 3-fold concentrated CM. The control medium (EBM-2 medium containing standard supplements without cells) was incubated under the same conditions and concentrated as above.

### 2.4. Irradiation

Irradiation was conducted with X-rays at a voltage of 120 kV and a 2 mm aluminum filter using an Isovolt Titan 160 X-ray machine (GE Sensing & Inspection Technologies, Ahrensburg, Germany). The focus-field distance was 21 cm. Irradiation doses of 2 Gy or 5 Gy were achieved at a dose rate of 2 Gy per minute. 0 Gy was utilized as control.

### 2.5. Flow Cytometry for Analysis of Apoptosis and Necrosis

After the irradiation and treatment of the cells with ADSC CM (including controls), cells including the supernatant were harvested. Cells were washed, resuspended in 200 µL of Ringer solution, and stained with 10 µL of a 1:1 mixture of Annexin V-APC (BD Biosciences, Heidelberg, Germany) and 7-amino-actinomycin D (7-AAD; BD Biosciences) for 30 min on ice. Cell suspensions were transferred to 96-well plates to investigate apoptosis and necrosis via the Cytoflex flow cytometer (Cytoflex, Beckman Coulter, Brea, CA, USA). Excitation at 660/10 nm was used to measure Annexin V-APC stained cells; excitation at 546 nm was used to measure 7-AAD stained cells. For data evaluation, FlowJo™ Analysis Software v10 (FlowJo LCC, BD Biosciences, Ashland, OR, USA) was used. Annexin V-APC-positive and 7AAD-negativ cells were defined as apoptotic cells, and Annexin V-APC-positive and 7AAD-positive cells as necrotic cells. Double-negative cells (Annexin V-APC-negative and 7AAD-negative) were defined as viable cells. Cells without any staining were used for a negative control, and cells treated with 56° C for 20 min served as positive control. Flow cytometry was performed in technical triplicate and in three independent experiments.

### 2.6. Invasion and Transmigration Assay

Invasion and transmigration assays were performed in a 24-well plate using transwell inserts with a pore size of 8 µm (ThinCert™, Greiner Bio-One GmbH, Frickenhausen, Germany), whereby transwells for invasion assays were coated with 2.4 mg/mL collagen type I from bovine skin (Sigma-Aldrich). A total of 72 h after irradiation, MCF-10A cells were harvested, and 1 × 10^5^ cells were seeded with 300 µl reduced mixed co-culture medium into the transwells. A total of 700 µl of ADSC CM or corresponding control medium was filled into the lower chamber. The cells were incubated for 8 h at 37 °C and 5% CO_2_. Thereafter, the transwell inserts were washed with PBS, followed by fixation with ice-cold methanol and staining with 1 µg/mL 4′,6-diamidino-2-phenylindole (DAPI) for 10 min (Life technologies). A PBS-coated cotton swab was used in twisting motions to remove the remaining cells in the inner part of the transwells. For counting transmigrated and invaded cells in the four quadrants of each transwell, an Olympus IX83 microscope (cellSens Software, Olympus Corporation, Tokio, Japan) was used for 40-fold magnification. Transmigration and invasion assays were performed in technical triplicate and in three independent experiments. Transmigrated or invaded cells, respectively, were semi-automatically measured with Fiji Is Just ImageJ (Fiji) [37].

### 2.7. Quantitative Real-Time PCR

Expression of selected cytokines, extracellular matrix (ECM) markers, and mesenchymal markers was analyzed at the mRNA level. Therefore, RNA was extracted using the RNeasy Mini Kit (Qiagen, Hilden, Germany). QuantiTect Reverse Transcription Kit with a DNase I incubation (Qiagen) was used for the reverse transcription of RNA into cDNA. Quantitative real-time PCR was conducted with a Bio-Rad CFX96 Real-Time PCR detection system (Bio-Rad Laboratories, Hercules, CA, USA) with the SsoAdvanced™ Universal SYBR^®^ Green Supermix (Bio-Rad Laboratories). The kits were used according to the manufacturers’ recommendations. Detected transcript levels were normalized to the housekeeping genes Tyrosine 3-monooxygenase/tryptophan 5-monooxygenase activation protein, zeta (*YWHAZ*), and Glyceraldehyde-3-phosphate dehydrogenase (*GAPDH*) using the 2^−ΔΔCT^-method. Samples were tested in technical triplicate and PCR was performed in three independent experiments. Primer sequences are specified in Table 1.

### 2.8. Statistics

Data are presented as mean ± standard deviation. All assays were performed in three replicate experiments. Statistical analysis was performed using the Mann–Whitney U test; the asymptotic significance was used (SPSS v.21.0 Software/IBM, Armonk, NY, USA). Figures were created with GraphPad Prism version 8.3.0 (La Jolla, CA, USA). A *p*-value < 0.05 was considered significant.

## 3. Results

### 3.1. ADSC CM Stimulated the Transmigration, Invasion, and Decreased Survival of MCF-10A Cells after Irradiation with 5 Gy 

**Figure 1:**
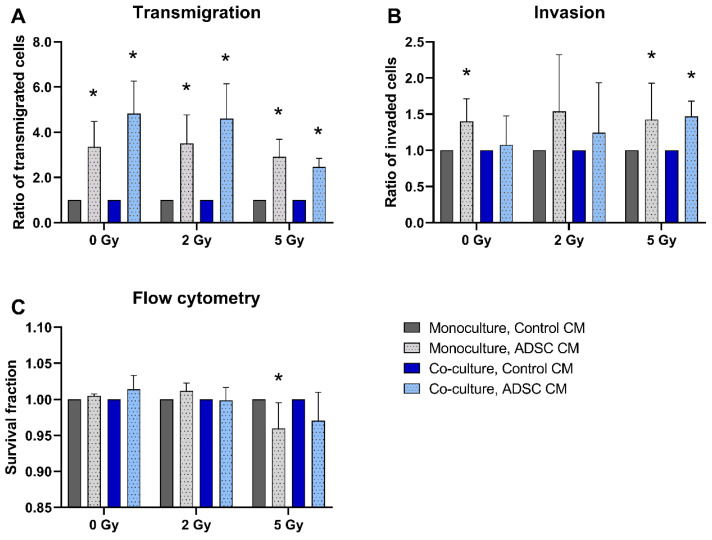
Effect of adipose-derived stem cell conditioned medium (ADSC CM) on the transmigration (**A**), invasion (**B**), and cell survival (**C**) of MCF-10A cells. MCF-10A cells were cultured as a mono- or co-culture with fibroblasts; irradiated with 0, 2, or 5 Gy; and treated with either ADSC CM or control CM. Data from the ADSC CM treatment group were normalized to the corresponding group treated with control CM (control = 1). (**A**–**C**) Columns show the ratio of transmigrated cells (**A**), invaded cells (**B**), and the survival fraction (**C**) compared to the control group (y-axis) in mono- and co-cultured cells in varying irradiation doses treated with ADSC CM or control CM (x-axis). *n* = 3 replicate experiments, and values are presented as mean ± SD. * *p* < 0.05, and the ADSC CM was compared to the control CM (Mann–Whitney U-test).

ADSC CM significantly stimulated the transmigration of MCF-10A in monoculture, as well as in co-culture with MRC-5 (*p* = 0.037; Figure 1A). In the 5 Gy irradiated cells, this effect decreased compared to non-irradiated samples or samples irradiated with 2 Gy. Additionally, the invasion ability of MCF-10A cells increased after treatment with ADSC CM in MCF-10A monoculture with 0 Gy irradiation and in both monoculture and co-culture after irradiation with 5 Gy (*p* = 0.037; Figure 1B). Apoptosis and necrosis analysis using flow cytometry detected lower cell survival after treatment with ADSC CM within the 5 Gy irradiation group (*p* = 0.037; Figure 1C). 

For analyzing the effects of ADSC CM, data of cells treated with ADSC CM were normalized to the corresponding mono- or co-culture group that were treated with control CM.

### 3.2. Co-Cultivation with Fibroblasts had a Small Effect on Transmigration and No Effect on the Invasion and Survival of MCF-10A Cells

**Figure 2:**
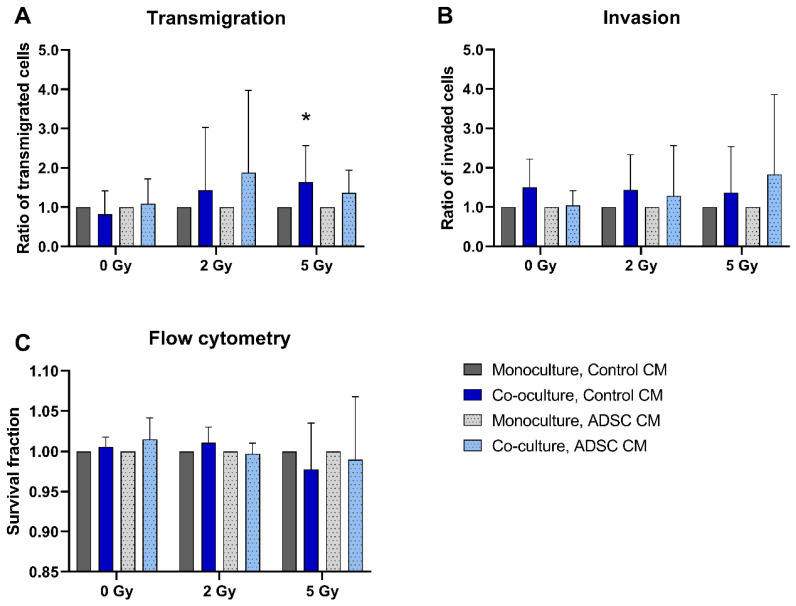
Effect of co-cultured fibroblasts on the transmigration (**A**), invasion (**B**), and cell survival (**C**) of MCF-10A cells compared to monocultured MCF-10A cells. MCF-10A cells were cultured as mono- or co-culture with fibroblasts; irradiated with 0, 2, or 5 Gy; and treated either with adipose-derived stem cell conditioned medium (ADSC CM) or control CM. Data from the co-culture group were normalized to the corresponding monoculture group (control = 1). (**A**–**C**) Columns show the ratio of transmigrated cells (**A**), invaded cells (**B**), and the survival fraction (**C**) compared to the control group (y-axis) in mono- and co-cultured cells in varying irradiation doses and treated with ADSC CM or control CM (x-axis). *n* = 3 replicate experiments, and values are presented as mean ± SD. * *p* < 0.05, and the co-culture was compared to the monoculture (Mann-Whitney U-Test).

The transmigration rate of co-cultured and 5 Gy-irradiated MCF-10A cells treated with control CM was significantly higher compared to monocultures (*p* = 0.037; Figure 2A). Co-culture with fibroblasts had no significant effect on MCF-10A invasion or cell survival (Figure 2B,C).

### 3.3. Irradiation of Mono- and Co-Cultures Decreased Transmigration and Cell Survival and Stimulated the Invasion Rates of MCF-10A Cells

**Figure 3:**
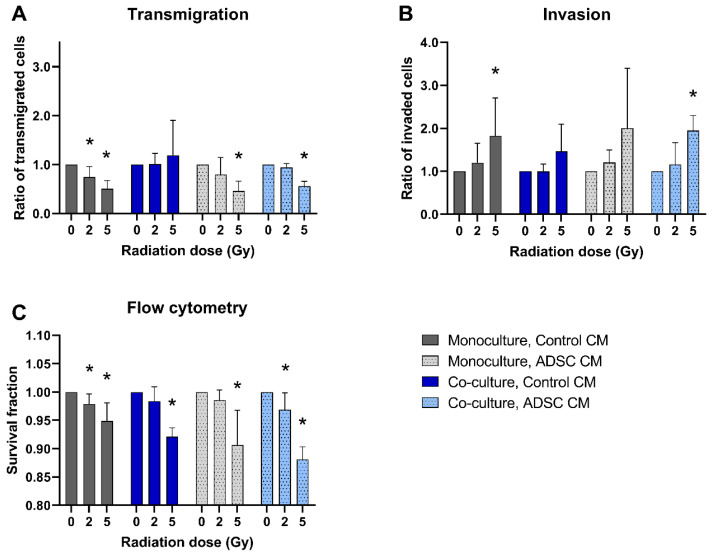
Effect of radiation on the transmigration (**A**), invasion (**B**), and cell survival (**C**) of MCF-10A cells compared to non-irradiated cells. MCF-10A cells were cultured as mono- or co-culture with fibroblasts; irradiated with 0, 2, or 5 Gy, and treated either with adipose-derived stem cell conditioned medium (ADSC CM) or control CM. Irradiation with 0 Gy was set 1 for each group. (**A**–**C**) Columns show the ratio of transmigrated cells (**A**), invaded cells (**B**), and the percentage of live cells (C; y-axis) in varying radiation doses and for mono- and co-cultured cells treated with ADSC CM or control CM (x-axis). *n* = 3 replicate experiments, and values are presented as mean ± SD. *: *p* < 0.05, and 5 Gy and 2 Gy were compared to 0 Gy (Mann–Whitney U-test).

A total of 5 Gy irradiation inhibited the transmigration of MCF-10A cells in monocultures and in co-cultures treated with ADSC CM compared to non-irradiated cells (*p* = 0.037; Figure 3A). In contrast, the invasion of monocultured MCF-10A cells treated with control CM and of co-cultured MCF-10A cells treated with ADSC CM was significantly increased after irradiation with 5 Gy (*p* = 0.037; Figure 3B). Irradiation decreased the cell survival of MCF-10A cells significantly, whereby the surviving fraction was lower after irradiation with the higher irradiation dose (Figure 3C).

### 3.4. Treatment with ADSC CM Induced Alterations in the Gene Expression Profile in Co-cultured and Irradiated MCF-10A Cells

**Figure 4:**
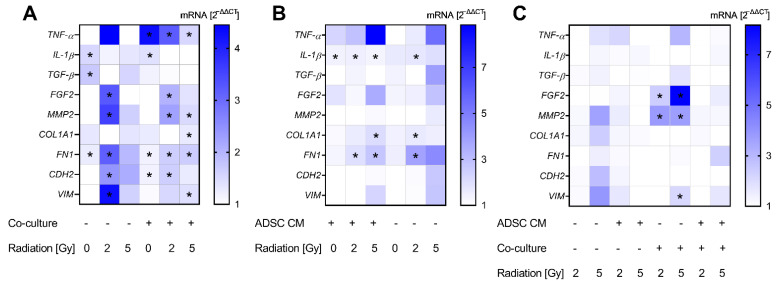
Alterations in gene expression profiles at the mRNA level in MCF-10A cells. The heatmaps show mean relative mRNA expression in MCF-10A cells compared to *GAPDH/YWHAZ* and to the corresponding control group, *n* = 3 replicate experiments. (**A**) Shows the effect of treatment with conditioned medium (CM) of adipose-derived stem cells (ADSC) normalized to treatment with control CM. (**B**) Presents the effect of co-cultivation with fibroblasts normalized to monocultures. The effect of irradiation is demonstrated in (**C**), whereby irradiation with 2 Gy and 5 Gy is normalized to 0 Gy. Values were calculated using the 2^−ΔΔCT^ method, 2^−ΔΔCT^ of control = 1. White fields: 2^−ΔΔCT^ < 1; * *p* < 0.05, increased compared to the corresponding control (Mann–Whitney U-test). *TNF-α*: tumor necrosis factor alpha, *IL-1β*: interleukin-1β, *TGF-β*: transforming growth factor-β, *FGF2*: fibroblast growth factor-2, *MMP2*: matrix metallopeptidase 2, *COL1A1*: collagen type I alpha 1 chain, *FN1*: fibronectin 1, *CDH2*: cadherin-2/N-cadherin, *VIM*: vimentin.

For investigating changes in the gene expression of MCF-10A cells, we tested the expression of several cytokines, ECM markers, and mesenchymal markers. Thereby, alterations after treatment with ADSC CM could be mainly observed in co-cultivated and irradiated MCF-10A cells. Results are summarized in Figure 4A. Co-cultured MCF-10A cells showed an increase in the expression of tumor necrosis factor alpha (*TNF-α*), independently of the applied radiation dose (*p* = 0.037). Further, the expression of fibroblast growth factor-2 (*FGF2*) was enhanced in 2 Gy-irradiated monocultured and co-cultured MCF-10A cells after treatment with ADSC CM compared to treatment with control CM (*p* = 0.037). mRNA levels of interleukin-1β (*IL-1β*) were up-regulated significantly after treatment with ADSC CM in monocultured and co-cultured, non-irradiated MCF-10A cells (*p* = 0.037). Transforming growth factor-β (*TGF-β*) appears to play a minor role and was only enhanced in monocultured, non-irradiated MCF-10A cells after treatment with ADSC CM (*p* = 0.037). Regarding ECM markers, we observed an ADSC CM-induced upregulation of matrix metallopeptidase 2 (*MMP2*) in 2 Gy-irradiated monocultured and in 2 and 5 Gy-irradiated co-cultured MCF-10A cells. Further, ADSC CM stimulated the expression of collagen type I alpha 1 chain (*COL1A1*) in 5 Gy-irradiated co-cultured MCF-10A cells (*p* = 0.037), suggesting an influence of ADSC on ECM remodeling. Moreover, treatment with ADSC CM induced an upregulation of mesenchymal markers. Thereby, the expression of fibronectin 1 (*FN1*) was upregulated in irradiated, as well as in non-irradiated, MCF-10A cells, whereas the mRNA levels of N-cadherin (*CDH2*) and vimentin (*VIM*) were only enhanced in irradiated cells, indicating the involvement of radiation-induced pathways. 

Data on co-cultures were also normalized to the corresponding conditions in monoculture to analyze the effect of co-cultivation with fibroblasts (Figure 4B). Thereby, we found a small, but significant, increase in *IL-1β* expression in co-cultured MCF-10A cells in the ADSC CM treatment group (*p* = 0.037). Moreover, *COL1A1* and *FN1* were enhanced in irradiated co-cultured MCF-10A cells. 

Normalizing the data of 2 Gy- and 5 Gy-irradiated groups to the corresponding non-irradiated cells revealed a 2 and 5 Gy irradiation-induced upregulation of *FGF2* and *MMP2* in co-cultured MCF-10A cells treated with control CM (*p* = 0.037; Figure 4C). Furthermore, 5 Gy irradiation induced an enhanced expression of *VIM* in co-cultured and non-irradiated MCF-10A cells (*p* = 0.037).

### 3.5. In MRC-5 Cells, Co-Cultured MRC-5 Cells Revealed an Upregulation of IL-1β, and Irradiation of Co-Cultured MRC-5 Cells Showed a Higher Expression of TGF-β

**Figure 5:**
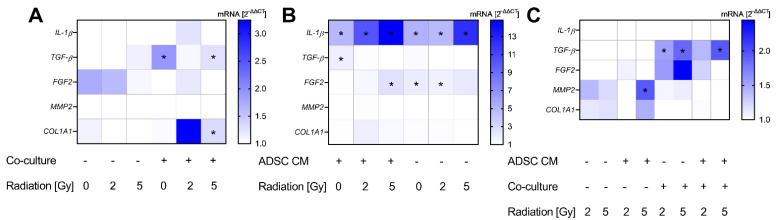
Alterations in gene expression profiles at the mRNA level in MRC-5 cells. The heatmaps show mean relative mRNA expression in MRC-5 cells compared to *GAPDH/YWHAZ* and to the corresponding control group, *n* = 3 replicate experiments. (**A**) Shows the effect of treatment with conditioned medium (CM) of adipose-derived stem cells (ADSC) normalized to treatment with control CM. (**B**) Presents the effect of co-cultivation with MCF-10A cells normalized to fibroblast monocultures. The effect of irradiation is demonstrated in (**C**), whereby irradiation with 2 Gy and 5 Gy is normalized to 0 Gy. Values were calculated using the 2^−ΔΔCT^ method, 2^−ΔΔCT^ of control = 1. White fields: 2^−ΔΔCT^ < 1; * *p* < 0.05, increased compared to the corresponding control (Mann–Whitney U-test). *IL-1β*: interleukin-1β, *TGF-β*: transforming growth factor-β, *FGF2*: fibroblast growth factor-2, *MMP2*: matrix metallopeptidase 2, *COL1A11*: collagen type I alpha 1 chain.

For a gene expression analysis of MRC-5 cells, the selected cytokines and ECM marker according to recent results were examined. Altogether, we observed a minor effect from ADSC CM and irradiation on the gene expression of fibroblasts, apart from *TGF-β*. Results are summarized in Figure 5. Treatment with ADSC CM revealed an increased expression of *TGF-β* in non-irradiated and 5 Gy-irradiated and co-cultured MRC-5 cells (*p* = 0.037; Figure 5A). Further, we observed an enhanced expression of *COL1A1* in co-cultured and 5 Gy-irradiated fibroblasts after treatment with ADSC CM (*p* = 0.037; Figure 5A).

The co-cultivation of MRC-5 with MCF-10A cells showed an upregulation of *IL-1β* (*p* = 0.037) with an up-to-14-fold increase in 5 Gy-irradiated cells and independently from treatment with ADSC CM (Figure 5B). Moreover, co-cultivation stimulated the expression of *FGF2* in 5 Gy-irradiated fibroblasts treated with ADSC CM and in 0 and 2 Gy-irradiated fibroblasts treated with control CM (*p* = 0.037). *TGF-β* was only upregulated in the non-irradiated and ADSC CM group. However, irradiation evoked higher *TGF-β* mRNA levels in co-cultured fibroblasts (Figure 5C). Whereas no stimulatory effect of ADSC CM and co-culture per se could be observed in *MMP2* expression, irradiation with 5 Gy revealed higher mRNA levels of *MMP2* in monocultured and ADSC CM-treated fibroblasts compared to the non-irradiated cells (*p* = 0.037), supporting an influence of irradiation on MMP activation and ECM digestion.

## 4. Discussion

Despite contradictory data regarding the safety of lipotransfer, the application of lipotransfer after BCT in breast cancer patients is an already widespread procedure for reconstructing volume deficits of the diseased breast. Concerns particularly arise with regard to the regeneration of tissue at an anatomic region where cancer has been treated. The question whether lipotransfer induces or accelerates the development of a subclinical tumor, locoregional disease recurrence, or tumor dissemination has not yet been clarified. Further experimental and standardized clinical studies are urgently needed to answer this question from different perspectives and to draw reliable conclusions. As irradiation of the residual breast tissue is an integral part after BCT in most of the cases, the effect of irradiation should not be neglected. Further, the tumor microenvironment was described as an essential part of malignant transformation. Thus, investigations on the influence of lipotransfer should include the tumor microenvironment. In this study, we analyzed the effects of lipotransfer on irradiated and non-irradiated MCF-10 cells in monoculture or in co-culture with fibroblasts as the main cellular component of the tumor microenvironment. Moreover, alterations in gene expression in fibroblasts were examined. 

In the present study we observed a strong stimulatory effect of ADSC CM on the transmigration of mammary epithelial cells. Further, the invasion of MCF-10A cells was stimulated by ADSC CM, though to a lesser extent compared to transmigration capability. Additionally, the mesenchymal cell markers *FN1*, *CDH2*, and *VIM* were upregulated in irradiated MCF-10A cells after treatment with ADSC CM compared to control CM. *FN1* participates in cell growth, migration, and wound healing under homeostatic conditions. In mammary epithelium, the targeted deletion of *FN* leads to the growth restriction of branching ducts and deficient alveologenesis [38]. However, it has also been found to be increased in several types of cancers and to play a central role in tumorigenesis [39]. *CDH2* was correlated with upregulated motility and invasion in human breast epithelial and breast carcinoma cell lines [40,41]. Similarly, the enhanced expression of *VIM* was associated with the increased migration and invasion of cancer cells [42]. Moreover, the upregulation of *FN1*, *CDH2*, and *VIM* is commonly associated with the epithelial–mesenchymal transition (EMT) in breast cancer and also in mammary epithelial cells [43]. The gain of mesenchymal cell markers and the increased migratory and invasive capabilities of MCF-10A cells observed in the present study represent features of EMT [44]. MCF-10A represents a breast cell line with intrinsic plasticity sharing stem-cell-like characteristics and was shown to be able to undergo EMT or EMT-like phenotypic changes [45]. EMT is evident for epithelial plasticity during embryogenesis, tissue homeostasis, and tissue repair or wound healing, respectively [46]. On the other hand, previous data suggested the involvement of EMT in early or later phases of breast cancer development [47]. It is still equivocal whether EMT in mammary epithelial cells leads to the production of stems cells [46]. 

We further observed an increase in the expression of cytokines in MCF10A cells after treatment with ADSC CM. Thereby, the ADSC CM treatment of MCF-10A cells in co-culture with fibroblasts lead to an enhanced expression of *TNF-α* in MCF-10A cells. Previous studies reported a potential cytotoxic and anti-tumor effect, as well as an important role in breast cancer progression and the local recurrence of *TNF-α* [48,49]. Thereby, *TNF-α* was suggested to be able to induce EMT in healthy breast epithelial cells like MCF-10A cells and malignant breast cells [50,51]. Further, treatment with ADSC CM led to a higher expression of *FGF2* in irradiated MCF-10A cells. *FGF2* plays an important role in fibroblast ECM synthesis and remodeling. In organotypic 3D co-cultures, FGF2 signaling was shown to increase the fibroblast-induced branching of mammary epithelium [52]. Reduced activity in FGF signaling under physiological conditions inhibits epithelial branching. However, the upregulation of *FGF* was shown to disrupt cell polarity and to induce cell proliferation, migration, and invasion capability, resembling molecular processes during breast cancer development [53]. Thus, the enhanced expression of cytokines might promote tumor development in healthy breast tissue. Moreover, an upregulation of cytokines in mammary epithelial cells by ADSC in the course of breast reconstruction might be relevant in case of residual adjacent malignant cells persisting after BCT that might be activated by those cytokines.

Treatment with ADSC CM had only a minor effect on the expression of *IL-1β* of MCF-10A cells. However, the co-cultivation of mammary epithelial cells with fibroblasts treated with ADSC CM revealed a higher expression of *IL-1β* compared to cells cultivated in monocultures. Previous findings demonstrated a correlation of upregulated *IL-1β* with neoplasm initiation and development. Thereby, an overexpression of *IL-1β* was found in both epithelial cells and in fibroblasts [54]. In the present study, we also found an upregulation of *IL-1β* in mammary epithelial cells and in fibroblasts in co-cultures compared to their monocultures, whereby the effect was more pronounced in fibroblasts, especially after 5 Gy irradiation. These findings suggest an interplay of mammary epithelial cells and fibroblasts that stimulates *IL-1β* expression mainly in fibroblasts under “physiological” conditions without ADSC and without irradiation as well as after 2 and 5 Gy-irradiation. However, the stimulatory effect on *IL-1β* expression of fibroblasts was enhanced by irradiation, indicating the involvement of radiation-induced pathways. Moreover, whereas treatment with ADSC CM and co-cultivation with fibroblasts had only a slight effect on the expression of *TGF-β* in MCF-10A cells, the irradiation of co-cultivated fibroblasts showed an upregulation of *TGF-β* in those cells. TGF-β was shown to induce a more aggressive cancer phenotype in breast cancer cells. Additionally, it is considered a potent EMT-promoting cytokine and was suggested to stimulate the neoplastic progression of transformed epithelial cells [55]. However, it was recently reported that TGF-β alone scarcely induces EMT [56]. Interestingly, a previous study could show that radiation-induced signaling pathways provoke heritable phenotypes that might be involved in carcinogenesis and that a single exposure to irradiation can sensitize mammary epithelial cells to undergo TGF-β-mediated EMT [57]. These findings support the relevance of irradiation in the pathophysiology of breast cancer development and progression. A further observation induced by irradiation in the present study is the enhanced invasion capability of MCF-10A. Whereas transmigration rates were inhibited after irradiation, MCF-10A cells showed increased invasion rates after irradiation with 5 Gy. No significant stimulation was observed after 2 Gy irradiation, which is in concordance with previous findings [58], indicating that higher irradiation doses are necessary to evoke this effect. As already expected and reported in earlier studies, irradiation decreased the survival rates of MCF-10A cells [58]. Although a regenerative effect is ascribed to ADSC, the ADSC CM decreased the surviving fraction of MCF-10A cells after irradiation with 5 Gy. 

The ECM has been attributed not only a role in normal breast development and differentiation but has also been attributed an evident function in breast cancer tumorigenesis due to imbalances in the ECM remodeling processes [59]. MMPs are involved in the reorganization of the tumor stroma and are supposed to have a decisive role in breast cancer invasion and metastasis [60]. Thereby, MMP2 and MMP9 can stimulate cell proliferation and angiogenesis and have been attributed a role in early-stage tumorigenesis and cancer progression [61,62]. COL1A1, as a further essential component of the ECM, was also correlated with breast cancer progression and metastasis [63]. In breast cancer stroma, *COL1A1* was identified as one of the most promising genes for tumor detection and treatment [64]. In the present study, treatment with ADSC CM enhanced the expression of *MMP2* and *COL1A1* in irradiated MCF-10A cells. Moreover, in fibroblasts, treatment with ADSC CM promoted the mRNA levels of *COL1A1* in irradiated cells, and irradiation enhanced the mRNA levels of *MMP2*. Interestingly, we could not observe any effect on *MMP2* or *COL1A1* in non-irradiated groups, suggesting an essential role of irradiation in inducing those upregulations of ECM markers. An upregulation of collagen genes might also contribute to a radiation-associated secondary breast cancer [65].

We also want to discuss several limitations of this study. Firstly, breast tissue consists of many additional components with a heterogeneous cell population, including i.a. endothelial cells, adipocytes, or immune-competent cells that were not addressed in this study. Therefore, the study is only of limited significance and only partially transferable into clinical practice. Nevertheless, the inclusion of further cell types would lead to more complex data in which the detected effects would be more difficult to assign to the respective subgroup. Additionally, the interval between irradiation and lipotransfer is much longer in patients so that the effect of irradiation in vitro might not depict the clinical reality. Another limitation is that direct cell–cell interactions between ADSC, mammary epithelial cells and fibroblasts were not taken into account. However, the data of the present study indicate that the use of lipotransfer should still be evaluated critically. Our data suggest EMT-like changes in the epithelial mammary breast cell line that might contribute to improved tissue regeneration. On the other hand, those changes might also induce the neoplastic transformation of the breast. Further, residual adjacent malignant cells persisting after BCT might be activated by ADSC. Thus, the benefits of lipotransfer after BCT should be critically weighed against its possible risks for the affected patients. Subgroup analyses in the past showed that some conditions are associated with higher recurrence rates after lipotransfer [66]. Thereby, e.g., breast cancer subtype, high-grade histology, age, Ki-67 expression, and the number of positive axillary nodes seem to play an important role [67,68]. Therefore, it is of special importance to identify high-risk patients and to discuss lipotransfer in a personalized approach for each patient.

More standardized and prospective clinical studies that include a considerable number of patients who underwent BCT are needed to further elucidate that issue on the clinical side. Moreover, there is a need of further in vitro and in vivo studies to provide a better understanding of the underlying cellular mechanisms. Thereby, translating experimental studies to clinical reality is difficult. Future experimental studies should include the surrounding microenvironment, e.g., within the context of 3D experiments. In addition, the effect of lipotransfer on the remaining breast cancer cells, including different breast cancer subtypes, surrounded by cancer-associated fibroblasts should be examined more closely.

## 5. Conclusions

Treatment with ADSC CM promoted the transmigration and invasion of MCF-10A cells and stimulated the expression of mesenchymal markers, suggesting EMT-like changes in mammary epithelial cells. Further, ADSC CM, in combination with irradiation, revealed increased mRNA levels of ECM markers in mammary epithelial cells and fibroblasts. We further observed the enhanced expression of tumor-promoting cytokines, which might play an important role in the presence of microscopic residual tumor foci after BCT. The data of the present study suggest a careful consideration of the need for lipotransfer after BCT of the individual patient.

## Figures and Tables

**Table 1:** Primer sequences.

Gene	Forward	Reverse
*FGF2*	CCACCTATAATTGGTCAAAGTGGTT	TCATCAGTTACCAGCTCCCCC
*TNF-Alpha*	TGGGATCATTGCCCTGTGAG	GGTGTCTGAAGGAGGGGGTA
*IL1B*	GCTCGCCAGTGAAATGATGG	GGTGGTCGGAGATTCGTAGC
*TGFB1*	CATGGAGGACCTGGATGCC	TCCTGAAGACTCCCCAGACC
*COL1A1*	GCTCTTGCAACATCTCCCCT	CCTTCCTGACTCTCCTCCGA
*MMP2*	GCCGTGTTTGCCATCTGTTT	AGCAGACACCATCACCTGTG
*FN1*	GAGAAGTATGTGCATGGTGTCAG	AATACTTCGACAGGACCACTTGA
*VIM*	AATCCAAGTTTGCTGACCTCTC	GTCTCCGGTACTCAGTGGACTC
*CDH2*	TCAATGACAATCCTCCAGAGTTTA	TGATCCTTATCGGTCACAGTTAGA
*YWHAZ*	ATGAGCTGGTTCAGAAGGCC	AAGATGACCTACGGGCTCCT
*GAPDH*	TCCACCCATGGCAAATTCCA	TTCCCGTTCTCAGCCTTGAC

*FGF2*: fibroblast growth factor-2, *TNF-Alpha*: tumor necrosis factor alpha, *IL1B*: interleukin-1β, *TGFB1*: transforming growth factor-β, *COL1A1*: collagen type I alpha 1 chain, *MMP2*: matrix metallopeptidase 2, *FN1*: fibronectin 1, *VIM*: vimentin, *CDH2*: cadherin-2/N-cadherin, *YWHAZ*: tyrosine 3-monooxygenase/tryptophan 5-monooxygenase activation protein, zeta, *GAPDH*: glyceraldehyde-3-phosphate dehydrogenase.

## Data Availability

The datasets generated during and/or analyzed during the current study are available from the corresponding author on reasonable request.
